# Triple M Syndrome After Immune Checkpoint Inhibition: A Rare and Life-Threatening Triad of Myocarditis, Myositis, and Myasthenia Gravis

**DOI:** 10.7759/cureus.96514

**Published:** 2025-11-10

**Authors:** Eman Albastaki, Rishu Rishu, Yati Tun, Alend Abdullah, Shakya Bhattacharjee

**Affiliations:** 1 Acute Medicine, The Dudley Group NHS Foundation Trust, Dudley, GBR; 2 General Internal Medicine, The Dudley Group NHS Foundation Trust, Dudley, GBR; 3 Cardiology, South Warwickshire University NHS Foundation Trust, Warwick, GBR; 4 Neurology, The Dudley Group NHS Foundation Trust, Dudley, GBR

**Keywords:** immune checkpoint inhibitor, myasthenia gravis, myocarditis, myositis, triple m syndrome

## Abstract

Triple M syndrome is a well-known complication of pembrolizumab and other immune checkpoint inhibition (ICI) therapy that entails three components, namely, myocarditis, myositis, and myasthenia gravis, which can present independently or as part of an overlap syndrome.

We present the case of an 80-year-old patient with stage 3C non-small cell lung carcinoma who complained of bulbar and ocular symptoms after the second cycle of pembrolizumab. Based on the integrated clinical picture and comprehensive biochemical markers, a provisional diagnosis of triple M syndrome was established. The patient was initiated on immunomodulatory therapy consisting of intravenous methylprednisolone and intravenous immunoglobulin (IVIG), administered over a five-day course. Despite first-line immunotherapy, the patient's condition deteriorated within four days of hospital admission. Plasmapheresis was considered but deemed unfeasible due to being too unstable to transfer to the tertiary centre to provide this. Given the lack of therapeutic response, the multidisciplinary team, in agreement with the patient's family, elected to transition the goals of care toward palliation and symptom-focused management.

This case report, together with previous reports, underscores the critical importance of maintaining a high index of suspicion for triple M syndrome and initiating prompt intervention in patients receiving ICI therapy. Early identification and timely management are essential for optimizing clinical outcomes and are pivotal in preventing mortality.

## Introduction

Lung adenocarcinoma is a subtype of non-small cell lung cancer accounting for approximately 40% of all lung cancer cases [1]. Evidence shows that pembrolizumab improved survival in patients with highly programmed death ligand 1 (PD-L1) expressing advanced non-small cell lung cancer [1]. Programmed death 1 (PD-1), an immune checkpoint receptor, is primarily expressed on activated T and B cells [2]. Pembrolizumab is a high-affinity, humanized, IgG4, highly selective monoclonal antibody against PD-1, which is a type of immune checkpoint inhibitor (ICI). The introduction of immunotherapy has transformed cancer treatment by harnessing the body's immune system to attack tumour cells. However, growing recognition of immunotherapy-related adverse events has highlighted that these reactions can sometimes be severe or even life-threatening. Although system-specific immune-related adverse events (irAEs) are well-described, emerging evidence points to the presence of overlap syndrome. Triple M syndrome is a rare but well-known complication of ICI therapy that consists of three components: myocarditis, myositis, and myasthenia gravis. They can occur independently or as an overlap syndrome, with a mortality rate recorded as high as 38% [3]. 

In this case, we report a patient with a background of stage 3C non-small cell lung carcinoma who presented with symptoms of overlap triple M syndrome, after four weeks of having the second cycle of pembrolizumab. We aim to emphasize the cruciality of high suspicion, early detection, and intervention to improve the clinical outcome. 

## Case presentation

We present the case of an 80-year-old man with a background of chronic kidney disease, renal cell carcinoma, type 2 diabetes mellitus, and hypertension. He was diagnosed recently with right upper and medial lobe lung adenocarcinoma and commenced on pembrolizumab to control the disease and extend life.

Four weeks after his second chemotherapy cycle, the patient presented to the emergency department following a general practitioner (GP) referral due to abnormal blood results indicating hyperkalaemia. On further assessment, he also reported slurred speech and bilateral ptosis. A contrast-enhanced computed tomography (CT) scan of the head was performed, which showed no acute intracranial pathology; therefore, the patient was discharged. However, he returned to the emergency department one week later with progressively worsening bilateral ptosis, dysphonia, slurred speech, and new-onset shortness of breath and dysphagia.

On bedside neurological examination, the patient had bilateral fatigable ptosis, weak neck flexion, hoarseness of voice with loss of cough reflex, and reflexes globally reduced but present symmetrically. The patient was reviewed by the neurology team on the first day of admission, who diagnosed this as pembrolizumab-induced myasthenic crisis, and was started on intramuscular neostigmine 2.5 mg three times a day (TDS), intravenous methylprednisolone, and intravenous immunoglobulin (IVIG) over five days (total dose 2 gm/kg) as per the discussion with the neuromuscular team at a tertiary centre. The immunotherapy was decided to be stopped. 

**Figure 1:**
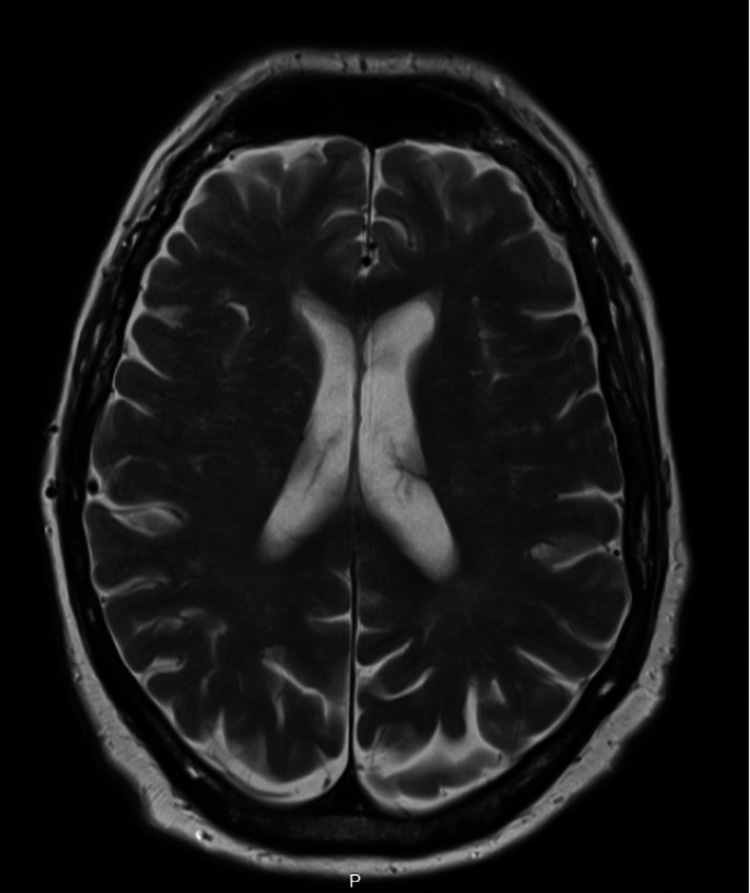
T2-weighted axial MRI of the brain image The MRI of the brain image showed no space-occupying lesion or brain metastasis as possible causes for the patient's symptoms. MRI: magnetic resonance imaging

The neurology team suggested possible myocarditis and myositis, as supported by elevated creatinine kinase (1478 IU/L), alanine aminotransferase (233 IU/L), and lactate dehydrogenase (474 IU/L). Troponin levels were also checked, and this was 800, 700, and 400 ng/mL (Table 1). A bedside electrocardiogram (ECG) showed no new changes, and an echocardiogram revealed no left ventricular systolic dysfunction. Electromyography (EMG) and spirometry were part of the investigation plan, but they were not done in view of the patient's instability. The patient had a magnetic resonance imaging (MRI) of his brain, which ruled out space-occupying lesions (Figure 1). Although the patient was on the abovementioned treatment, his symptoms were worsening. He developed complete ptosis and aphasia and was unable to tolerate oral intake; therefore, a nasogastric tube was inserted. It was planned for the patient to have a trial of plasmapheresis, but this facility was not available in our trust, and the patient was too unstable to be shifted to a tertiary hospital. He developed an oxygen requirement and subsequently went into type 2 respiratory failure (Table 2) on day 4 of admission.

**Table 1:** Biochemical markers at presentation to the emergency department eGFR: estimated glomerular filtration rate; ALT: alanine aminotransferase; CRP: C-reactive protein; CK: creatine kinase; LDH: lactate dehydrogenase

Biochemical marker	At presentation	Normal range
Sodium	142 mmol/L	135-145 mmol/L
Potassium	5.5 mmol/L	3.5-5 mmol/L
eGFR	43 mL/min/1.73 m^2^	>90 mL/min/1.73 m^2^
ALT	233 U/L	40-50 U/L
Bilirubin	7 µmol/L	5-21 µmol/L
Albumin	27 g/L	30-50 g/L
CRP	27 mg/L	<5 mg/L
CK	1478 U/L	38-174 U/L
Troponin	807.4 ng/L	<10 ng/L
LDH	474 U/L	140-280 U/L
Haemoglobin	118 g/L	135-175 g/L
White blood cell count	13.7×10⁹/L	4.5-11.0×10⁹/L

**Table 2:** Arterial blood gas on deterioration pH: potential of hydrogen; pO2: partial pressure of oxygen; pCO2: partial pressure of carbon dioxide; HCO3: bicarbonate

Arterial blood gas	At deterioration	Normal range
pH	7.26	7.35-7.45
pO2	8.2 kPa	10.0-13.0 kPa
pCO2	9.82 kPa	4.7-6.0 kPa
HCO3	32.9 mmol/L	22-28 mmol/L
Lactate	<1.0 mmol/L	0.5-2.2 mmol/L

The critical care team discussed with the family, who agreed that invasive mechanical ventilation would not be in the patient's best interest. Thus, it was decided not to admit the patient to the intensive care unit and keep him for ward-based care. He was started on symptom-based treatment, and the focus was shifted to end-of-life care. Unfortunately, the patient passed away the following day. 

## Discussion

As per the literature, the onset of signs and symptoms for each or either of the components of triple M syndrome usually occurs as soon as the first cycle is started, while in the case of myocarditis, it tends to start within 12 weeks of initiation of the therapy [4]. In our case, the patient was showing symptoms correlating with the picture of myasthenia gravis after receiving the second cycle of ICI therapy and later developed biochemical evidence of myositis and myocarditis. 

A high index of suspicion for triple M syndrome should be maintained in any patient receiving ICI therapy, given its potentially life-threatening nature and the critical importance of early intervention [4-6]. In our case, although the patient exhibited symptoms suggestive of myasthenia gravis following the initial treatment cycles, further evaluation for the possibility of triple M was not undertaken at that stage, possibly attributed to limited awareness of ICI-induced triple M syndrome. 

The investigations for the early diagnosis of triple M components, that is, myocarditis (raised troponin T, creatine kinase-MB, echocardiogram, ECG) and myositis (elevated creatine kinase, liver transaminases, aldolase, lactate dehydrogenase, C-reactive protein, erythrocyte sedimentation rate), are highly suggestive of the aforementioned [3-5]. However, for myasthenia, clinical assessment and neurophysiological studies overrule the biomarkers (anticholinesterase (anti-ACR) and muscle-specific tyrosine kinase (anti-MUSK) antibodies) in comparison to non-ICI-induced myasthenia gravis [4,6-8]. 

Gold standard investigations for the diagnosis of myocarditis are cardiac biopsy and cardiac MRI. For myositis and myasthenia gravis, muscle biopsy and EMG are definitive for diagnosis [3,6]. 

In our case, the patient demonstrated elevated troponin levels, creatine kinase, liver transaminases, and lactate dehydrogenase. Although a definitive diagnosis could not be established through specific investigations due to the patient's clinical instability and inability to be transferred to a tertiary care centre, there remained a high clinical suspicion for components of triple M based on the presenting symptoms and available biomarker profile. 

The management of triple M requires a multidisciplinary approach, with immunosuppression being the main therapeutic strategy; however, there is currently no consensus regarding the optimal type, dosage, or duration of immunosuppressive therapy [6,9]. It was proven that patients on mono-steroid therapy showed less clinical improvement [10]. Multiple studies have shown that early, combined treatment with corticosteroids, IVIG, and plasmapheresis is associated with improved outcomes of triple M and a reduced incidence of paradoxical exacerbation of myasthenic symptoms compared to steroid monotherapy [3,4,7,11-14]. In one study discussing a series of four cases of triple M syndrome, all patients were successfully discharged from the intensive care unit following the implementation of an aggressive treatment approach utilizing this combined therapy [6]. In some cases, within this study, immunomodulatory therapy demonstrated benefit in patients who did not respond adequately to the initial combined treatment regimen [6]. In another study, the use of mycophenolate and rituximab as a second-line treatment after using the combined therapy was also recommended [3]. 

In our case, the patient was started on neostigmine intramuscularly 2.5 mg TDS, intravenous methylprednisolone, and IVIG over five days (total dose 2 gm/kg) on his admission to the hospital. Given the patient's clinical presentation and the suspicion of triple M syndrome, early consideration of plasmapheresis may have been beneficial. Additionally, transfer to a tertiary care centre could have facilitated access to advanced therapies such as plasmapheresis and other immunomodulatory treatments. However, these were not possible due to the patient's rapid deterioration and clinical instability. 

Literature suggests that bilevel positive airway pressure (BiPAP) is recommended in the management of myasthenic crisis, as it provides continuous pressure support that helps reduce the risk of atelectasis and upper airway collapse [6]. None of the supported ventilation types was implemented in our case, as it was not deemed appropriate for the patient, given his poor prognosis. 

## Conclusions

This case highlights the critical importance of early recognition and maintaining a high index of suspicion for triple M syndrome, both of which are essential for timely intervention and are key factors in improving patient outcomes. Clinicians should ensure regular follow-up for patients receiving ICI therapy and maintain a high index of suspicion for possible immune-related adverse effects associated with such treatments. If any element of triple M syndrome is identified, a comprehensive evaluation should be undertaken to investigate the other components, enabling early diagnosis and prompt management. Early aggressive treatment may play a crucial role in improving clinical outcomes, slowing disease progression, and reducing mortality.
